# Supplementary material for: Type 2 and interferon inflammation regulate SARS-CoV-2 entry factor expression in the airway epithelium
Source: Nat Commun. 2020 Oct 12;11:5139. doi: 10.1038/s41467-020-18781-2 (PMC7550582; doi:10.1038/s41467-020-18781-2)
Supplement: Supplementary file 1 — Supplementary Information [file 41467_2020_18781_MOESM1_ESM.pdf]

Supplementary Information for:

Type 2 and interferon inflammation strongly regulate SARS-CoV-2 related gene expression in the airway epithelium

Authors/Affiliations

Satria P. Sajuthi[1#], Peter DeFord[1#], Yingchun Li[1], Nathan D. Jackson[1], Michael T. Montgomery[1], Jamie L. Everman[1], Cydney L. Rios[1], Elmar Pruesse[1], James D. Nolin[1], Elizabeth G. Plender[1], Michael E. Wechsler[2], Angel CY Mak[3], Celeste Eng[3], Sandra Salazar[3], Vivian Medina[4], Eric M. Wohlford[3,5], Scott Huntsman[3], Deborah A. Nickerson[6,7,8], Soren Germer[9], Michael C. Zody[9], Gonçalo Abecasis[10], Hyun Min Kang[10], Kenneth M. Rice[11], Rajesh Kumar[12], Sam Oh[3], Jose Rodriguez-Santana[4], Esteban G. Burchard[3,13], Max A. Seibold[1,14,15*]

[1]Center for Genes, Environment, and Health, National Jewish Health, Denver, CO, USA; [2]Department of Medicine, National Jewish Health, Denver, CO, USA; [3]Department of Medicine, University of California San Francisco, San Francisco, CA; [4]Centro de Neumología Pediátrica, San Juan, Puerto Rico; [5]Division of Pediatric Allergy and Immunology, University of California San Francisco, San Francisco, CA; [6]Department of Genome Sciences, University of Washington, Seattle, WA, USA; [7]Northwest Genomics Center, Seattle, WA, USA; [8]Brotman Baty Institute, Seattle, WA, USA; [9]New York Genome Center, New York City, NY; [10]Center for Statistical Genetics, University of Michigan, Ann Arbor, MI, USA; [11]Department of Biostatistics, University of Washington,

Seattle, WA, USA; [12]Ann and Robert H. Lurie Children's Hospital of Chicago, Department of Pediatrics, Northwestern University, Chicago, IL; [13]Department of Bioengineering and Therapeutic Sciences University of California San Francisco, San Francisco, CA; [14]Department of Pediatrics, National Jewish Health, Denver, CO, USA; [15]Division of Pulmonary Sciences and Critical Care Medicine, University of Colorado-AMC, Aurora, CO, USA; [#]these authors contributed equally to this work, *correspondence: seiboldm@njhealth.org

Supplementary Figure 1

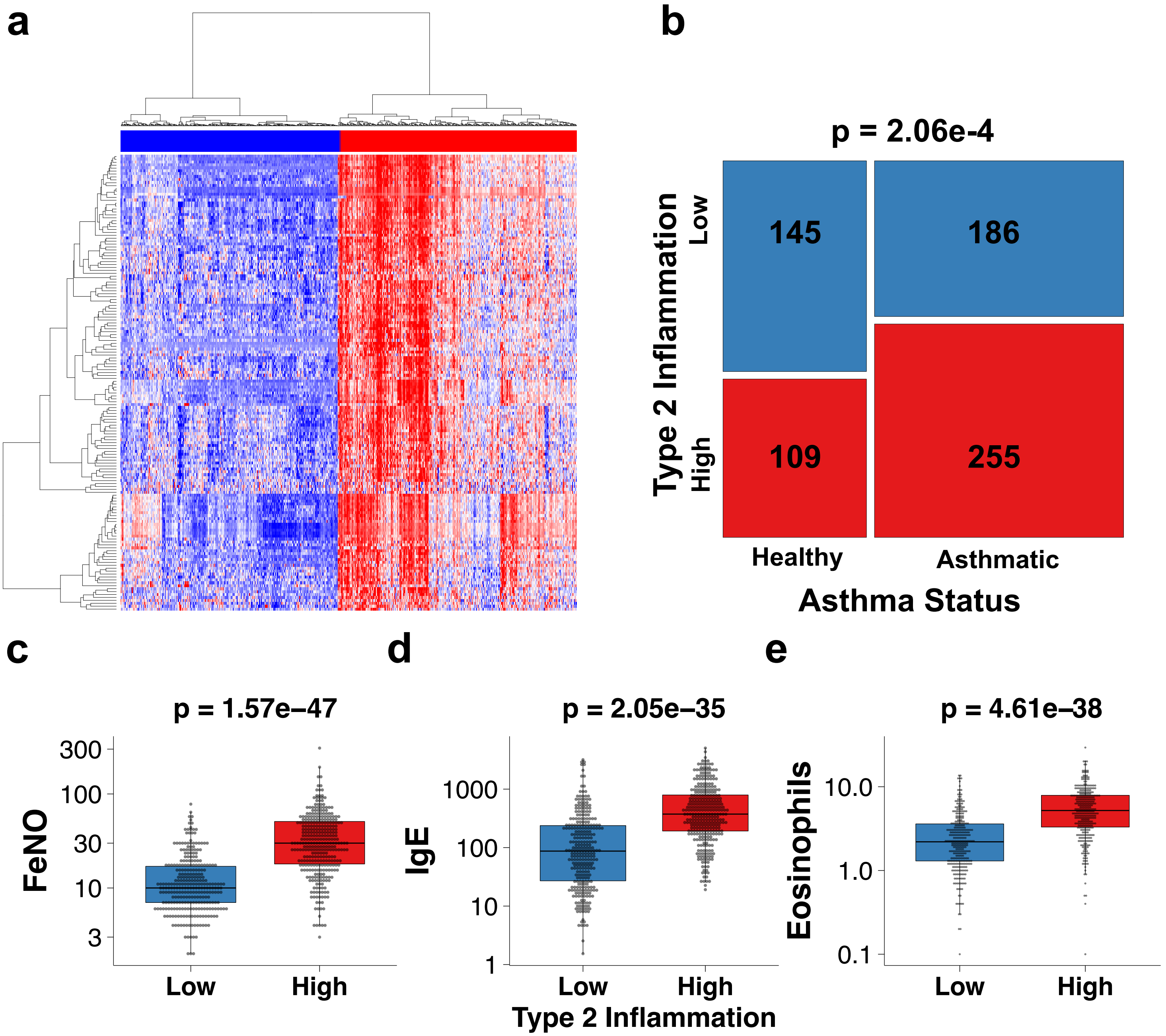

Supplementary Figure 1. Identification of T2-high subjects and association with clinical traits

(a) Clustering of subjects by expression of T2 biomarker network (saddle brown) genes identifies T2-high and T2-low groups. Heatmap of the T2 biomarker network genes across all clustered subjects. The colorbars at the top of the heatmap correspond to the T2 clusters. The blue and red bars identify T2-low and T2-high subjects, respectively.

(b) Distribution of T2-status assignments based on asthma status. T2 status is associated with asthma status. Association testing between T2 status and asthma status was performed with a two-sided χ^2 test.

(c-e) Box plots of atopy associated clinical variable levels by T2-status. P-values are generated from a two-sided Wilcoxon-rank-sum test.

(c) Fractional exhaled nitric oxide in parts per billion (FeNO) by T2-status. (n; T2-high=336, T2-low=296)

(d) Total serum IgE levels by T2-status. (n; T2-high=327, T2-low=300)

(e) Whole blood eosinophil levels by T2-status. (n; T2-high=356, T2-low=324)

Box centers give the median, upper and lower box bounds correspond to first and third quartiles, and the upper/lower whiskers extend from the upper/lower bounds up to/down from the largest/smallest value, no further than 1.5 × IQR from the upper/lower bound (where IQR is the inter-quartile range). Data beyond the end of whiskers are plotted individually

Supplementary Figure 2

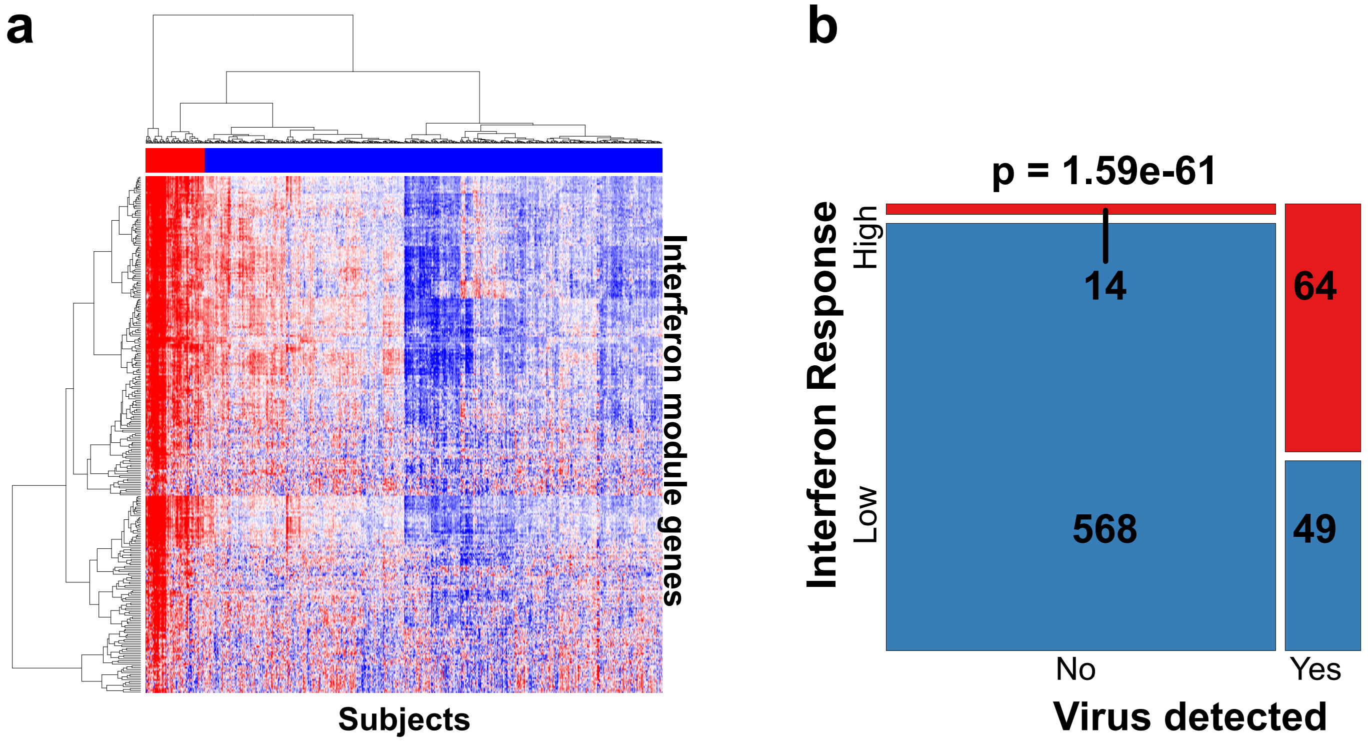

Supplementary Figure 2. Identification of interferon-high subjects and association with viral infection status

(a) Clustering of subjects by expression of interferon response network genes (tan) identifies interferon-high and interferon-low groups. Heatmap of the tan network genes across all clustered subjects. The colorbars at the top of the heatmap correspond to the interferon clusters. The blue and red bars identify interferon-low and interferon-high subjects, respectively.

(b) Distribution of interferon-status assignments based on viral infection status. Interferon-high status is associated with viral infection status. Association testing between interferon status and asthma status was performed with a two-sided χ^2 test.

Supplementary Figure 3

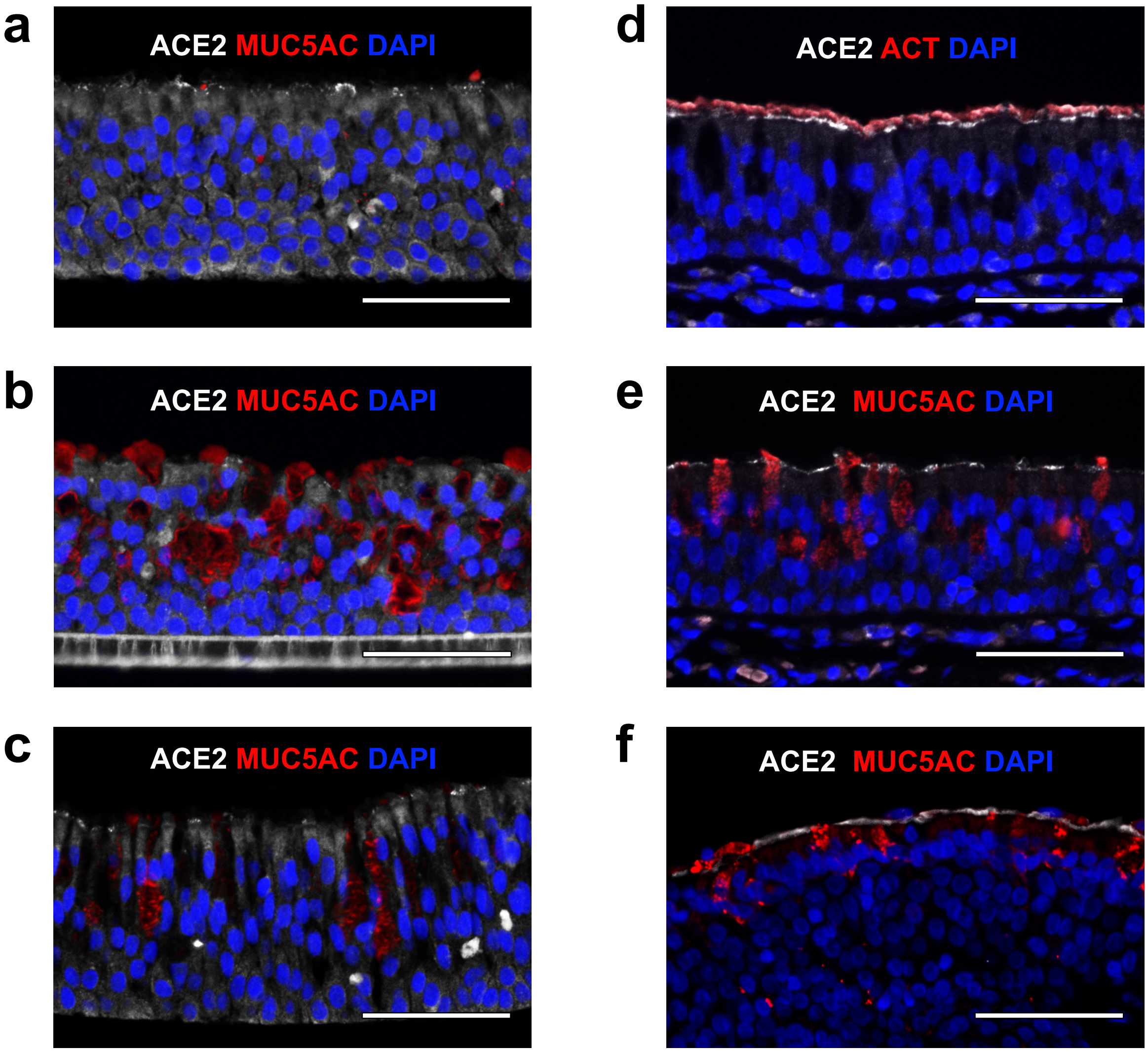

Supplementary Figure 3. ACE2 protein expression and localization by immunofluorescence staining

(a) to (c) Immunofluorescence staining of in vitro ALI nasal airway epithelial cultures derived from mock (a), IL-13 treated (5days of 10ng mL^{-1} IL-13; b), and HRV-A infected (24hr post-infected; c) epithelium. Representative images of goblet cells (MUC5AC; red) and ACE2 positive (white) cells. The nuclei were counterstained with DAPI (blue). Scale bars: 60 μm. (d-f) Immunofluorescence staining of in vivo trachea epithelium in two healthy donors. (d) Representative images of ciliated cells (ACT; red) and ACE2 positive (white) cells in Donor 2. (e-f) Representative images of goblet cells (MUC5AC; red) and ACE2 positive (white) cells in Donor 1 (e) and 2 (f). The nuclei were counterstained with DAPI (blue). Scale bars: 60 μm. All images were captured on the Echo Revolve R4 and images were cropped and resize using Affinity Designer. For each image, brightness and contrasts were uniformly adjusted relative to the brightest feature to balance exposure of each color channel.

Supplementary Figure 4

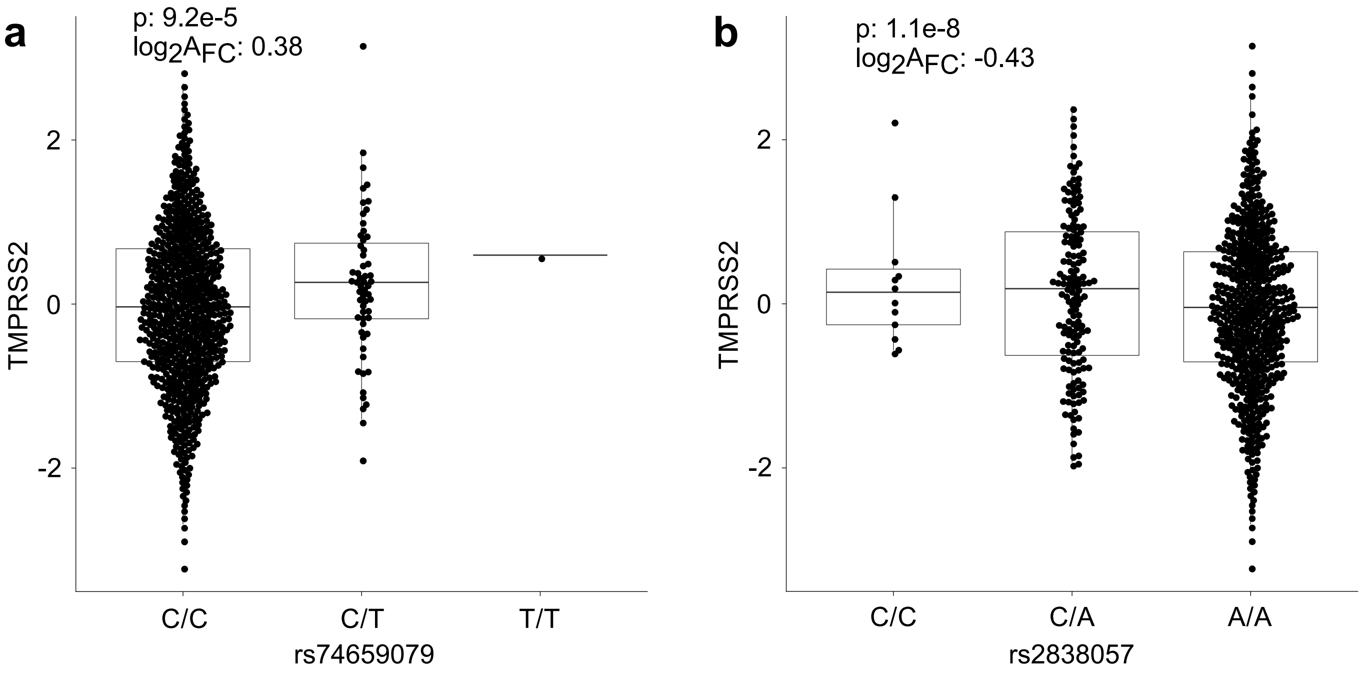

Supplementary Figure 4. Additional independent eQTLs for *TMPRSS2*

(a) Boxplot of *TMPRSS2* expression by genotype for eQTL variant rs74659079 (n; CC:622, CT=58, TT=1). eQTL p-values were obtained from testing the additive genotype effect on gene expression using the linear regression model implemented in FastQTL.

(b) Boxplot of *TMPRSS2* expression by genotype for eQTL variant rs2838057 (n; CC=12, CA=151, AA=517). eQTL p-values were obtained from testing the additive genotype effect on gene expression using the linear regression model implemented in FastQTL.

Box centers give the median, upper and lower box bounds correspond to first and third quartiles, and the upper/lower whiskers extend from the upper/lower bounds up to/down from the largest/smallest value, no further than 1.5× IQR from the upper/lower bound (where IQR is the inter-quartile range). Data beyond the end of whiskers are plotted individually

Supplementary Figure 5

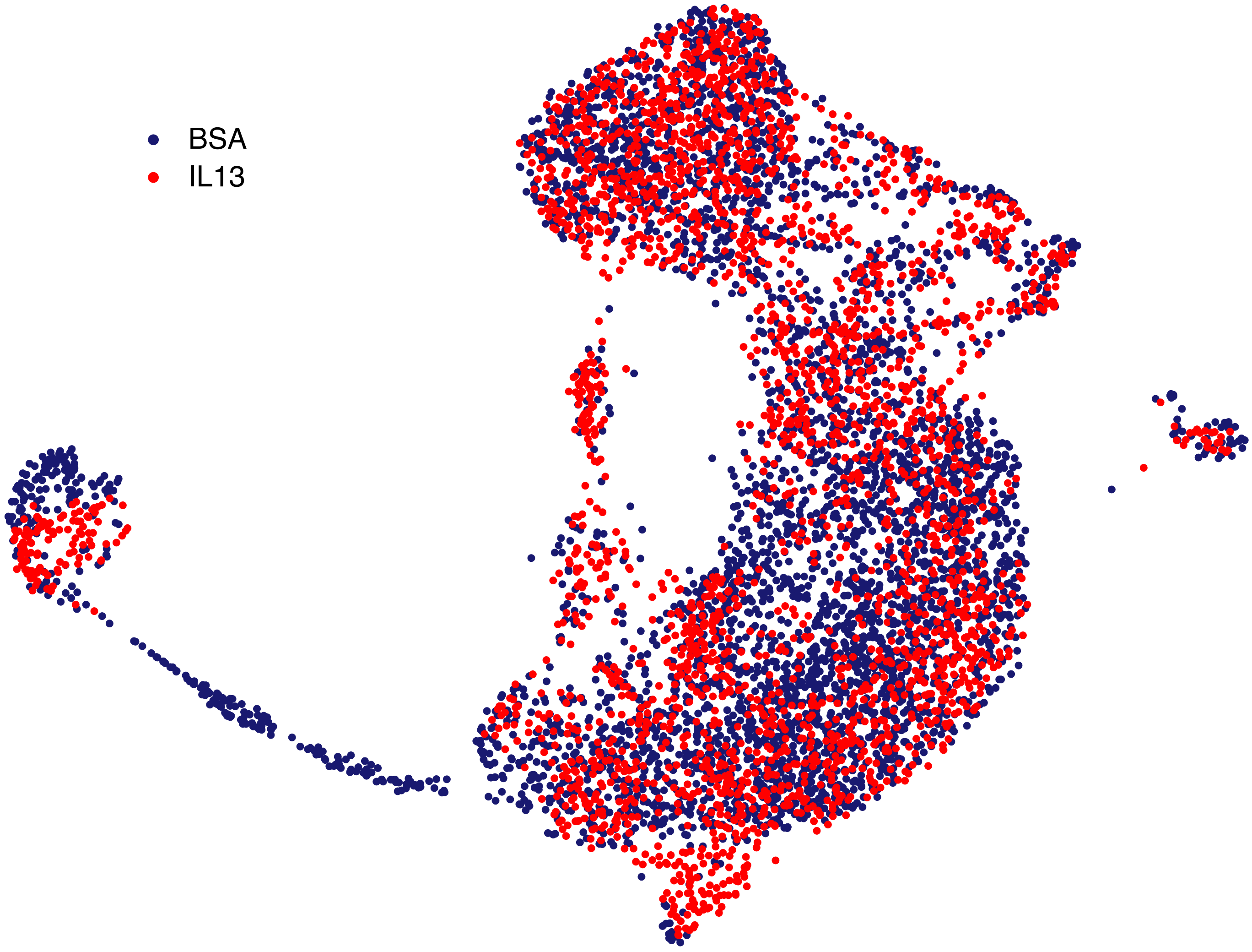

Supplementary Figure 5. UMAP of scRNA-seq data from chronic IL-13 culture experiment with cells colored by IL-13 or mock treatment status

Blue and red cells come from cultures stimulated with either mock or IL13 stimulus, respectively.
