# Supplementary material for: Type 2 and interferon inflammation regulate SARS-CoV-2 entry factor expression in the airway epithelium
Source: Nat Commun. 2020 Oct 12;11:5139. doi: 10.1038/s41467-020-18781-2 (PMC7550582; doi:10.1038/s41467-020-18781-2)
Supplement: Supplementary file 3 — Reporting Summary [file 41467_2020_18781_MOESM3_ESM.pdf]

nature research

Corresponding author(s): Max A. Seibold

Last updated by author(s): Aug 28, 2020

Reporting Summary

Nature Research wishes to improve the reproducibility of the work that we publish. This form provides structure for consistency and transparency in reporting. For further information on Nature Research policies, see our Editorial Policies and the Editorial Policy Checklist.

Statistics

For all statistical analyses, confirm that the following items are present in the figure legend, table legend, main text, or Methods section.

n/a	Confirmed	
☐	☒	The exact sample size (n) for each experimental group/condition, given as a discrete number and unit of measurement
☐	☒	A statement on whether measurements were taken from distinct samples or whether the same sample was measured repeatedly
☐	☒	The statistical test(s) used AND whether they are one- or two-sided<br>*Only common tests should be described solely by name; describe more complex techniques in the Methods section.*
☐	☒	A description of all covariates tested
☐	☒	A description of any assumptions or corrections, such as tests of normality and adjustment for multiple comparisons
☐	☒	A full description of the statistical parameters including central tendency (e.g. means) or other basic estimates (e.g. regression coefficient) AND variation (e.g. standard deviation) or associated estimates of uncertainty (e.g. confidence intervals)
☐	☒	For null hypothesis testing, the test statistic (e.g. F, t, r) with confidence intervals, effect sizes, degrees of freedom and P value noted<br>*Give P values as exact values whenever suitable.*
☒	☐	For Bayesian analysis, information on the choice of priors and Markov chain Monte Carlo settings
☒	☐	For hierarchical and complex designs, identification of the appropriate level for tests and full reporting of outcomes
☐	☒	Estimates of effect sizes (e.g. Cohen's d, Pearson's r), indicating how they were calculated

Our web collection on statistics for biologists contains articles on many of the points above.

Software and code

Policy information about availability of computer code

Data collection	None
Data analysis	Cell Ranger version 3.0 was used for in vivo and in vitro single cell preprocessing;<br>BWA-mem version 0.7.15 was used for mapping DNA sequence data;<br>GotCloud pipeline 1.17 was used for variant calling;<br>Eagle version 2.4 was used for haplotype phasing;<br>Skewer version 0.2.2 was used for trimming bulk RNA-seq data;<br>GSNAP version 2016_05_01 was used for mapping bulk RNA-seq data from GALA II;<br>HISAT2 version 2.1.0 was used for mapping bulk RNA-seq data from ALI cultures;<br>HTSeq-count version 0.9.1 was used for bulk-RNA seq data gene quantification;<br>DESeq2 version 1.22.2 was used to normalize bulk RNA-seq expression data;<br>edgeR version 3.28.0 was used to perform differential expression analysis between virus-infected and uninfected groups;<br>METASOFT version 2.0.0 was used to compute Cochran's Q statistics;<br>WGCNA version 1.68 was used to perform network analysis;<br>ADMIXTURE version 1.3 was used on genotype data to compute admixture factors;<br>PEER version 1.3 was used to generate PEER factors;<br>FastQTL version 2.184 modifed by GTEx was used to perform eQTL analysis (https://github.com/francois-a/fastqtl);<br>QTLTools version 1.1 was used to perform stepwise regression;<br>AFC version 180919 was used to calculate allelic fold change (https://github.com/secastel/aFC);<br>BBduk version 38.79 was used for quality and adapter trimming in the virus identification pipeline;<br>Bowtie2 version 2.4.1 was used for mapping in the virus identification pipeline;<br>Metaspades version 3.14.0 was used to assemble viral contig in the virus identification pipeline;

Samtools version 1.10 was used to calculate per contig read counts in the virus identification pipeline;
BlastN version 2.9.0 was used to match assembled viral contig to the human genome in the virus identification pipeline;
Seurat (R package) version 3.0 was used for all single cell dataset integration, clustering, visualization, and differential expression analysis;
Enrichr version 2017 (https://amp.pharm.mssm.edu/Enrichr/) was used for functional enrichment analysis;
Limma (R package) version 3.42.0 was used to perform differential expression analysis between virus-infected and uninfected groups;
Kallisto version 3.42.0 was used to quantify expression for eQTL analysis.
IPA (v01-16; content version: 51963813, release 2020-03-11) was used to investigate canonical pathways and upstream regulators;
Affinity Designer version 1.8.3 was used for image analysis
Custom code used has been deposited at https://github.com/seiboldlab/ACE2_TMPRSS2_Airway

For manuscripts utilizing custom algorithms or software that are central to the research but not yet described in published literature, software must be made available to editors and reviewers. We strongly encourage code deposition in a community repository (e.g. GitHub). See the Nature Research guidelines for submitting code & software for further information.

Data

Policy information about availability of data

All manuscripts must include a data availability statement. This statement should provide the following information, where applicable:

- Accession codes, unique identifiers, or web links for publicly available datasets
- A list of figures that have associated raw data
- A description of any restrictions on data availability

FASTQ files and normalized count matrix for GALA II study has been deposited to GEO GSE152004, the private access token for the reviewer is mjwvqswoljmbziz;
gnomAD database (version 2.1.1): https://gnomad.broadinstitute.org/;
GO Biological Process 2018 table [https://amp.pharm.mssm.edu/Enrichr/geneSetLibrary?mode=text&libraryName=GO_Biological_Process_2018];
GO Molecular Function 2018 table [https://amp.pharm.mssm.edu/Enrichr/geneSetLibrary?mode=text&libraryName=GO_Molecular_Function_2018];
GO Cellular Component 2018 table [https://amp.pharm.mssm.edu/Enrichr/geneSetLibrary?mode=text&libraryName=GO_Cellular_Component_2018];
Ligand Perturbations from GEO up table [https://amp.pharm.mssm.edu/Enrichr/geneSetLibrary?mode=text&libraryName=Ligand_Perturbations_from_GEO_up];
Kyoto Encyclopedia of Genes and Genomes 2019 Human table [https://amp.pharm.mssm.edu/Enrichr/geneSetLibrarymode=text&libraryName=KEGG_2019_Human];
Reactome 2016 table [https://amp.pharm.mssm.edu/Enrichr/geneSetLibrary?mode=text&libraryName=Reactome_2016];
Cell type marker gene sets were obtained from Supplemental Table S4 in Travaglini, et al. [https://www.biorxiv.org/content/biorxiv/early/2020/03/20/742320/DC1/embed/media-1.xlsx];
TOPMed freeze 8 variant calls are available from dbGaP accession phs000920.v2.p2 [https://www.ncbi.nlm.nih.gov/projects/gap/cgi-bin/study.cgi?study_id=phs000920.v4.p2];
NCBI NT BLAST database was downloaded May 1st 2020 [https://ftp.ncbi.nlm.nih.gov/blast/db/v5/];
NCBI Taxonomy was accessed May 25th [https://ftp.ncbi.nlm.nih.gov/pub/taxonomy/new_taxdump/new_taxdump.tar.gz];

Field-specific reporting

Please select the one below that is the best fit for your research. If you are not sure, read the appropriate sections before making your selection.

☒ Life sciences ☐ Behavioural & social sciences ☐ Ecological, evolutionary & environmental sciences

For a reference copy of the document with all sections, see nature.com/documents/nr-reporting-summary-flat.pdf

Life sciences study design

All studies must disclose on these points even when the disclosure is negative.

Sample size

For GALA II nasal transcriptomic study, no formal sample size calculation was done. The analysis from from this dataset was used to describe the heterogeneity of nasal airway transcriptomes. There are 695 donors with RNA-seq data.

For GALA II eQTL study, no formal sample size calculation was done. There are 681 donors with paired genotype and expression data which is larger than the sample size of any single tissue in GTEx.

For in vivo nasal brush scRNA-seq data, no formal sample size calculation was done. The sample size is 1. This data is used to identify the cell types expressing ACE2 and TMPRSS2. Although with sample size of one, our results largely mirrored the Human Cell Atlas (HCA) papers.

For scRNA-seq from paired 10 day IL13-stimulated and control tracheal ALI cultures, no formal sample size calculation was done. The sample size is 1. This data is used to study the IL13 effect on the expression of ACE2 and TMPRSS2 for each identified cell type which is based on many cells per group and is consistent with other results in the paper.

For the analysis of bulk RNA-seq data derived ACE2 and TMPRSS2 gene expression between paired IL13- stimulated and control nasal airway epithelial cultures, no formal sample size calculation was done. This analysis was based on paired cultures from 5 donors. This sample size was sufficient to identify differentially expressed genes.

For the analysis of bulk RNA-seq data derived ACE2 and TMPRSS2 gene expression between paired HRV-infected and control nasal airway epithelial cultures, no formal sample size calculation was done. This analysis was based on paired cultures from 5 donors. This sample size was sufficient to identify differentially expressed genes.

For the ACE2 immunofluorescence studies no sample size calculation was performed. The ACE2 staining quantification for the IL-13, HRV experiments were based on nasal airway epithelial cultures derived from a single donor, quantifying images of 20x objective fields, 12-13 fields per condition. The ACE2 staining quantification for the IL-13/DAPT differentiation experiments were based on nasal airway epithelial cultures derived from a single donor, quantifying images of 20x objective fields, 5-6 fields per condition. The number of fields counted by sufficient to see statistical differences in staining. These results were highly significant and consistent with the gene expression data.

For the ACE2 staining of in vivo tracheal airway epithelium, representative images are shown, no quantification or statistical tests were performed.

Data exclusions

For GALA II eQTL study, we excluded 14 donors that failed genotyping QC by TOPMED. Sample-level quality control included checks for pedigree errors, discrepancies between self-reported and genetic sex, and concordance with prior genotyping array data.

Replication

For GALA II nasal transcriptomics network study, no replication was performed, but results are robust due to the sample size, which is much larger than most published network studies of primary human tissues.

For GALA II eQTL study, no replication was performed, but results are robust due to the large sample size. There are 681 donors with paired genotype and expression data which is larger than the sample size of any single tissue in GTEx.

For in vivo nasal brush scRNA-seq data, no replication was performed. But our results largely mirror those of the Human Cell Atlas (HCA) papers.

For scRNA-seq from paired 10 day IL13-stimulated and control tracheal ALI cultures, no replication was performed. This data is used to study the IL13 effect on the expression of ACE2 and TMPRSS2 for each identified cell type which is based on many cells per group and is consistent with other results in the paper.

For the analysis of bulk RNA-seq data derived ACE2 and TMPRSS2 gene expression between paired IL13- stimulated and control nasal airway epithelial cultures, no replication was performed. But these results were highly significant and consistent with other results in the paper.

For the analysis of bulk RNA-seq data derived ACE2 and TMPRSS2 gene expression between paired HRV-infected and control nasal airway epithelial cultures, no replication was performed. But these results were highly significant and consistent with other results in the paper.

For the ACE2 immunofluorescence quantification studies no replication was performed. However, these results were highly significant and consistent with the gene expression data.

For the ACE2 staining of in vivo tracheal airway epithelium, representative images are shown, no quantification or statistical tests were performed.

Randomization

GALA II donors included in the network and eQTL analysis were obtained from recruitment as described. No randomization was performed in the analyses as it was not relevant, but the eQTL analysis controlled for relevant covariates (i.e.: sex, age, bmi, asthma status, and admixture estimate).

We randomly selected 5 GALA II donors for the IL-13/HRV, ACE2/TMPRSS2 experiments. These experiments were paired, with all treatments being applied to cultures from all donors, so randomized allocation of groups was unnecessary.

For other scRNA-seq and immunofluorescence experiments donors cells were selected from those available and randomization was not relevant.

Blinding

Most study results did not involve the combination of subjective decisions or a priori hypotheses, where classical blinding might be needed. Rather we conducted computational analyses and genome-wide statistical tests, then reported the results of these agnostic analyses. Therefore blinding was unnecessary.
For the ACE2 immunofluorescence quantification, the scientist was blinded to the groups for each image analyzed.

Reporting for specific materials, systems and methods

We require information from authors about some types of materials, experimental systems and methods used in many studies. Here, indicate whether each material, system or method listed is relevant to your study. If you are not sure if a list item applies to your research, read the appropriate section before selecting a response.

Materials & experimental systems

n/a	Involved in the study	
☐	☒	Antibodies
☐	☒	Eukaryotic cell lines
☒	☐	Palaeontology and archaeology
☒	☐	Animals and other organisms
☐	☒	Human research participants
☒	☐	Clinical data
☒	☐	Dual use research of concern

Methods

n/a	Involved in the study	
☒	☐	ChIP-seq
☒	☐	Flow cytometry
☒	☐	MRI-based neuroimaging

Antibodies

Antibodies used	ACT, host:mouse, supplier name: Sigma, catalog #: T6793, clone name: 6-11B-1, lot #: 108M4823V<br>MUC5AC, host: mouse, supplier name: Thermo Fisher, catalog #: MA1-38223, clone name: 45M1, lot #: SH2431615<br>KRT5, host: chicken, supplier name: BioLegend, catalog #: 905901; clone name: Poly9059, lot #: NA<br>ACE2, host: goat, supplier name: R&D Systems, catalog #: AF933, clone name: NA, lot #: HOK0320041<br>anti chicken-Alexa 488, host: donkey, supplier name: Jackson ImmunoResearch, catalog #: 703-545-155, clone name: NA, lot #: 137337<br>anti-mouse-Alexa 594, host: donkey, supplier name: Thermo Fisher, catalog #: a21203A21203, clone name: NA, lot #: 1990305<br>anti-goat-Alexa 647, host: donkey, supplier name: Thermo Fisher, catalog #: A21447, clone name: NA, lot #: 1841382
Validation	All antibodies have been validated by manufacturer as per the manufacturer websites

Eukaryotic cell lines

Policy information about cell lines

Cell line source(s)	NIH 3T3 Fibroblast cells were purchased from ATCC for use as feeder co-culture for primary human epithelial cells.
Authentication	No authentication was performed.
Mycoplasma contamination	No mycoplasma testing was performed.
Commonly misidentified lines<br>(See ICLAC register)	No commonly misidentified lines were used.

Human research participants

Policy information about studies involving human research participants

Population characteristics	The Genes-Environment & Admixture in Latino Americans study (GALA II) is an on-going case-control study of asthma in Latino children and adolescents. The study includes subjects with asthma and healthy controls of Latino descent between the ages of 8 and 21.<br><br>For in vivo nasal brush scRNA-seq data, we consented a 56 year old asthmatic subject, from which we collected nasal airway epithelial cells.<br><br>For scRNA-seq of the paired 10 day IL13-stimulated and control tracheal ALI cultures, a human tracheal sample was isolated from a single de-identified 57yo male non-smoking lung donor from the International Institute for the Advancement of Medicine (Edison, NJ), and Donor Alliance of Colorado.<br><br>For the IL13/HRV ACE2 immunofluorescence quantification studies, nasal derived ALI cultures from a GALA II asthmatic child were mock treated, IL-13 treated (5 days), or HRV-A16 and -C infected (harvested 24h post-infection).<br><br>For the IL13/DAPT ACE2 immunofluorescence quantification studies, nasal derived ALI cultures from a non-asthmatic child from the lung donor program were differentiated normally for 21 days, or differentiated for 21 days in the presence of IL-13 (daily treated), or DAPT (treated every other day) .<br><br>For the ACE2 staining of in vivo tracheal airway epithelium, tracheal tissues were obtained from 2 de-identified lung donors, all received from the International Institute for the Advancement of Medicine (Edison, NJ), and Donor Alliance of Colorado. One of the donors is the same donor used for the IL13/DAPT ACE2 IF quantification study.
Recruitment	GALA II Study subjects were recruited from community centers and clinics in the mainland U.S. and Puerto Rico (2006-present). Asthma case status was physician-diagnosed. Recruited subjects completed in-person questionnaires detailing medical, environmental, and demographic information<br><br>For in vivo nasal brush scRNA-seq data, we consented a 56 year old asthmatic subject at National Jewish Health<br><br>For scRNA-seq from paired 10 day IL13-stimulated and control tracheal ALI cultures, human tracheal samples was isolated from a single de-identified lung donor from the International Institute for the Advancement of Medicine (Edison, NJ), and Donor Alliance of Colorado.<br><br>For the IL13/HRV ACE2 immunofluorescence quantification studies, a nasal epithelial tissue was obtained from a GALA II asthmatic child (recruitment as describe above).<br><br>For the analysis of bulk RNA-seq data derived ACE2 and TMPRSS2 gene expression between paired IL13- and HRV-stimulated and control nasal airway epithelial cultures we used 5 GALA II donors (recruitment as describe above).<br><br>For the IL13/DAPT ACE2 immunofluorescence quantification studies, a nasal epithelial tissue was obtained from a non-asthmatic child received from the International Institute for the Advancement of Medicine (Edison, NJ), and Donor Alliance of Colorado.<br><br>For the ACE2 staining of in vivo tracheal airway epithelium, tracheal tissues were obtained from 2 de-identified lung donors, all received from the International Institute for the Advancement of Medicine (Edison, NJ), and Donor Alliance of Colorado. One of the donors is the same donor used for the IL13/DAPT ACE2 IF quantification study.<br><br>Our study of ACE2 and TMPRSS2 expression was conducted in Puerto Rican children. We caution that extrapolation of our results to other groups (e.g. adults and other racial groups) will require similar studies in those populations.
Ethics oversight	GALA II: University of California San Francisco (UCSF) IRB number 10–00889, Reference number 153543, NJH HS-2627<br><br>Nasal scRNA-seq: The Institutional Review Board (IRB) National Jewish Health (HS-3101)<br><br>Tracheal scRNA-seq: The National Jewish Health Institutional Review Board HS-3209 and HS-2240<br><br>ACE2 immunofluorescence quantification studies: The National Jewish Health Institutional Review Board HS-3209 and HS-2240

Note that full information on the approval of the study protocol must also be provided in the manuscript.
