# Supplementary material for: Type 2 and interferon inflammation regulate SARS-CoV-2 entry factor expression in the airway epithelium
Source: Nat Commun. 2020 Oct 12;11:5139. doi: 10.1038/s41467-020-18781-2 (PMC7550582; doi:10.1038/s41467-020-18781-2)
Supplement: Supplementary file 4 — Description of Additional Supplementary Files [file 41467_2020_18781_MOESM4_ESM.pdf]

Description of Additional Supplementary Files

File Name: Supplementary Data 1
Description: The number of ACE+ and/or TMPRSS2+ cells among epithelial and immune cell types.

File Name: Supplementary Data 2
Description: Gene Ontology and Cell Type enrichments for selected networks identified by WGCNA analysis. Enrichment p-values were obtained from one-sided hypergeometric test. Benjamini-Hochberg correction was used to control for false discovery rate.

File Name: Supplementary Data 3
Description: Correlation matrix for genes with WGCNA networks. Correlation value is based on Pearson product-moment correlation coefficient between normalized gene expression and network eigengene.

File Name: Supplementary Data 4
Description: Correlation matrix of WGCNA network eigengene values. The upper triangular of the matrix = Pearson correlation coefficients. The lower triangular of the matrix = p-values from a two-sided Pearson correlation test.

File Name: Supplementary Data 5
Description: Detected coronavirus species and prevalence for samples within the GALA II cohort.

File Name: Supplementary Data 6
Description: Detected virus species and prevalence for samples within the GALA II cohort.

File Name: Supplementary Data 7
Description: Table of differentially expressed genes between COV/ORV and uninfected individuals. Differential expression analysis was performed with limma. Benjamini-Hochberg correction was used to control for false discovery rate.

File Name: Supplementary Data 8
Description: Demographic table of GALA II study.

File Name: Supplementary Data 9
Description: Cell-type marker genes from the nasal brush sample. Differential expression analysis between cell populations was performed with two-sided Wilcoxon test. Bonferroni correction was used to control for family-wise error rate.

File Name: Supplementary Data 10
Description: Cell-type marker genes from the tracheal airway epithelial ALI samples. Differential expression analysis between cell populations was performed with two-sided Wilcoxon test. Bonferroni correction was used to control for family-wise error rate.
